# Public perceptions toward primary care in Kuwait: a cross-sectional study

**DOI:** 10.3389/fmed.2025.1643414

**Published:** 2025-12-10

**Authors:** Lulua Falah Alasousi, Ahmad T. Al-Sultan, Sara Mohammad Barakat AlHammouri, Teebah Adel Alhasan, Daliah Khaled Almurjan

**Affiliations:** 1 Ministry of Health of Kuwait, Sulaibikhat, Kuwait; 2 Department of Community Medicine and Behavioral Sciences, College of Medicine, Kuwait University, Kuwait City, Kuwait

**Keywords:** primary care, awareness, emergency department, family medicine, perception

## Abstract

**Introduction:**

An increase in the use of emergency departments in hospitals, influenced by multiple factors, is one of the main problems faced by healthcare leaders in Kuwait. A lack of confidence in general practice and low awareness of the severity of the situation highlight the need for reassurance by emergency specialists. Despite Kuwait’s substantial investment in its primary health care infrastructure, the patterns of its utilization have not been thoroughly examined. Therefore, we aimed to evaluate people’s perceptions of primary care services in Kuwait.

**Methods:**

This study was conducted between October 2023 and June 2024 in Kuwait. We used a convenience sampling technique to include individuals of the Kuwaiti population aged ≥18 years; we excluded primary care physicians. A paper-based, validated questionnaire was distributed to primary healthcare visitors. Overall, the sample comprised 655 participants.

**Results:**

Participants who were followed at primary care clinics and those who selected them as their first choice demonstrated greater awareness of the primary care role and higher scores regarding their impression of primary care doctors than those who did not. However, 53% of the participants believed that the emergency department was more important than a primary care center. Participants who acknowledged that the primary health care center is the first choice for treating medical problem were significantly more likely to have good impressions and good awareness (adjusted odds ratio [AOR] = 2.98; 95% CI: 1.77–5.02; *p* < 0.001) and (AOR = 5.09; 95% CI: 2.98–8.69; *p* < 0.001), respectively. Additionally, those who believe that the primary health care center holds more importance than the emergency department were more likely to have good impressions and awareness (AOR = 2.34; 95% CI: 1.68–3.25; *p* < 0.001) and (AOR = 2.64; 95% CI: 1.72–4.04; *p* < 0.001), respectively.

**Conclusion:**

Several factors influence people’s awareness of primary care. Therefore, stakeholders should promote awareness programs to increase the awareness of the value and role of primary care.

## 1 Introduction

Family medicine provides comprehensive primary care services to everyone; it is considered the cornerstone of the healthcare system and a fundamental element of each country’s public health policy. According to the World Health Organization (WHO), primary care is the most effective, efficient, and fair way to achieve increased health coverage (1). Despite remarkable improvements in healthcare systems across most developed and developing countries, health leaders still face many challenges.

One of the main challenges policymakers face is the rising number of patients visiting hospital emergency rooms for minor cases (2, 3). This increase in patient visits results in longer waiting times and hospital overcrowding, which may, in turn, affect the quality of emergency care services (4). In 2009, a systematic review revealed that the rate of inappropriate emergency service use among adults in the Kingdom of Saudi Arabia ranged from 20 to 40%. One reason for this unnecessary rise in emergency department use is the lack of confidence in primary care services (5, 6). Another study conducted in Jazan showed that only 56.3% of participants knew about the role of family physicians; however, 90.2% did not have a regular family physician, and 59.8% preferred seeing a specialist (7, 8). Therefore, increasing public awareness of the role of family physicians and primary care services is crucial for empowering family medicine practices.

Family medicine practice in Kuwait was established by the Ministry of Health (MOH) in 1983, with a steady increase in the number of primary care centers, reaching 115 across 6 governorates by 2023. Despite Kuwait’s significant investment in its primary health care infrastructure, utilization patterns have not been thoroughly examined in comparison to international standards (9). According to the WHO’s framework for resilient primary care systems, countries with similar economic profiles have increasingly shifted toward a community-based care model (10). However, Kuwait’s dependence on hospital services, especially for non-communicable diseases (NCDs), indicates a divergence from these trends. A 2022 study conducted in Kuwait found that hypertension was linked to a 75% increase in hospital admissions. Similarly, heart disease was associated with a fourfold rise in hospitalization rates (11). In a study in China involving 11,817 participants, each additional NCD was associated with a 38% increase in inpatient days (12). To our knowledge, no research has been conducted to assess people’s awareness of the role of family medicine in Kuwait. Therefore, we aimed to evaluate public perceptions of primary care in the country.

## 2 Materials and methods

This cross-sectional study was conducted in Kuwait from October 2023 to June 2024, covering all six Kuwaiti governorates: Ahmadi, Capital, Hawally, Farwaniya, Mubarak Al Kabeer, and Jahra. The research took place in primary care centers, with two centers selected from a list provided by the MOH. These centers were chosen because of their high number of Kuwaiti nationals. A paper-based questionnaire was distributed to Kuwaiti participants at two different government primary care centers in each sector, also selected from a list supplied by the MOH. We used convenience sampling for data collection due to limited resources.

According to the September 2023 census by the Public Authority for Civil Information, there were 880,314 Kuwaiti adults across all governorates. A sample size of 1,534 was estimated, assuming a 95% confidence interval (CI) (*Z* = 1.96), a 50% prevalence, and a 5% margin of error within each stratum. Sample size allocation was calculated by multiplying the population size of each governorate by the total sample size (1,534) and dividing by the total population (880,314). The samples for each governorate were divided equally between the two primary care centers, with 50% allocated to each center (Table 1).

**Table 1:** Population distribution by Kuwaiti governorate.

Governorate	Kuwaiti adult population	Sample size
Al-Ahmadi	179,674	313
Al-Asimah	181,398	315
Al-Farwaniyah	142,583	249
Al-Jahra	115,113	201
Hawally	154,961	270
Mubarak Al-Kabeer	106,585	186
Total	880,314	1,534

Ethical approval was obtained from the MOH (approval no. 2023/2371). The study included all Kuwaiti residents aged ≥18 years, as ~70% of Kuwait’s population actively uses the healthcare system. Kuwaiti citizens benefit from free healthcare services, which makes it easier to access primary care centers. Since these centers serve as the first point of contact, they contribute to higher utilization rates and distinct healthcare-seeking behaviors among non-Kuwaiti residents. Physicians from other medical specialties and MOH workers were included, while non-Kuwaiti residents and primary care physicians were excluded.

The validated Arabic version of the questionnaire was obtained from previous studies and comprises four sections (13, 14):

1. Demographic data (such as nationality, age, educational level, and governorate).
2. Public perception of the characteristics of primary care physicians.
3. Public knowledge of primary health care centers, their roles, and services compared to emergency departments.
4. Respondents’ attitudes toward family medicine, including their preferences for emergency departments, primary health care centers, or other specialist clinics for various health scenarios or complaints.

The questionnaire took about 2 min to complete. A team of trained family physicians, introduced the research idea to the participants and recorded their responses. Overall, 1,100 people participated in the study; 445 participants were excluded from the analysis because they did not finish the questionnaire. Therefore, 655 participants were included in the final analysis. The response rate was 43%.

### 2.1 Statistical analysis

The collected data were analyzed using IBM SPSS Statistics version 29 (IBM Corp., New York, USA). Descriptive statistics were used to present the data. All participants’ demographic variables are displayed as categorical variables and summarized as frequencies and percentages. For the assessment section, answers were scored from 0 to 4, corresponding to strongly disagree, disagree, neutral, agree, and strongly agree, respectively. The total score ranged from 0 to 60 for the 15 survey questions. A higher score indicated a greater level of the factor it represented. The Shapiro–Wilk test showed that the impression scores were not normally distributed. Therefore, the results were expressed as medians and interquartile ranges (IQRs) for each factor.

For the awareness of the role of primary health care centers and their services assessment, questions were scored by assigning scores of 1 for a “Yes” answer and 0 for a “No” or “Not sure” answer, resulting in a score range of 0–4 for each participant. We adopted the following cutoff points for scores: <50% as poor, 50–75% as fair, and ≥75% as good. We described awareness levels by calculating the frequency and percentage for each category.

To test the association between scores and demographics, the Mann–Whitney U tests, Kruskal−Wallis, and Spearman’s rank correlation tests were used to examine the relationship between impression scores of primary care doctors and patients’ demographics and other factors. Additionally, the Pearson Chi-square test, the Chi-square test for trend, and the Chi-square goodness-of-fit test were employed to assess differences in the frequency of awareness of the role of primary health care centers and their services across the factors of interest.

Two univariable logistic regression models were created. The first model was used to quantify the association between variables significantly associated with good impressions of primary care. An impression score was considered good if it fell between 75 and 100% of the total score And fair or poor if the score was below 75%. The second model quantified the association between variables significantly associated with good awareness of the primary care role. To identify variables that are independently and significantly (*p* < 0.05) associated with good impressions and good awareness, a multivariable logistic regression model was fitted to the data. The variables significantly (*p* < 0.05) and independently associated with good impressions and good awareness were retained in the model. Adjusted odds ratios (AORs) and their corresponding 95% CIs were used to interpret the model.

## 3 Results

A total of 655 participants completed the survey questionnaire. Among them, 57.6% were women and 42.4% were men. Most participants were followed up in primary care clinics (69.6%) and were aged 20–39 years (60.0%). The overall median unweighted and weighted impression scores were 46 (IQR = 19) and 3.1 (IQR = 1.3), respectively. As shown in Table 2, the statistical tests indicated a weak positive relationship between the impression and age (*p* = 0.047). However, there was a weak negative relationship between the governorate Kuwaiti adult population, sample size (Al-Ahmadi 179,674, 313; Al-Asimah 181,398, 315; Al-Farwaniyah 142,583, 249; Al-Jahra 115,113, 201; Hawally 154,961, 270; Mubarak Al-Kabeer 106,585, 186; and total 880,314, 1,534, 8) impressions, and educational level (*p* = 0.007).

**Table 2:** Relationship between sociodemographic variables and impressions about previous visits to primary care doctors.

Characteristics		Impressions		
		Median (IQR)	R/Z Score	*p*-value
Age (Years)	20–39	45 (18)	0.08	0.047
	40–60	46 (18)		
	More than 60	55 (19)		
Gender	Male	47 (18.3)	−1.53	0.127
	Female	45 (18)		
Education	High school	49 (17)	−0.11	0.007
	Diploma and other certificate	47.5 (18.8)		
	Bachelors	45 (18.3)		
	PhD/Master	43.5 (20)		
Governorate	Ahmadi	48.5 (22.3)	9.45	0.092
	Al Asimah	48 (16)		
	Farwaniya	42 (22)		
	Hawally	45 (16.5)		
	Jahra	44 (15)		
	Mubarak Al Kabeer	49 (19.3)		
Following up in primary care clinics	No	44 (19)	−3.46	0.001
	Yes	48 (18)		
The primary health care center is the first option for treating a medical problem	No	38 (20)	−5.71	<0.001
	Yes	48 (17.5)		
Whichever is more important	Emergency department	43 (16)	−6.68	<0.001
	Primary health care center	50 (16)		

IQR, interquartile range.

Participants who were regularly followed up in primary care clinics had higher impression scores than those who were not (*p* = 0.001). Additionally, the mean impression score of patients who selected a primary care center as their first choice to treat a medical issue was higher than that of patients who did not (*p* < 0.001) (Table 2).

None of the participants had a fair level of awareness of the primary healthcare centers’ role. The total number with poor and good awareness scores were 163 (24.9%) and 492 (75.1%), respectively. Regarding participants’ age, a significant linear relationship was observed between age and awareness level (*p* = 0.004); awareness increased with age. Similarly, as age increased, the proportion of poor awareness decreased. Additionally, a significant association was identified between the governorate and awareness levels (*p* = 0.011). Pairwise proportion tests for poor awareness showed no significant differences among the governorates. However, participants from Al Asimah governorate had significantly higher awareness scores than those from Mubarak Al Kabeer, Farwaniya, and Jahra (*p* < 0.001) (Table 3).

**Table 3:** Chi-square analysis of the comparison of the awareness about the role of primary care centers and service category, based on participants’ demographic and other factors of interest.

Characteristics		Awareness		
		Poor, *n* (%)	Good, *n* (%)	*p*-value
Age (Years)	20–39	113 (28.8)	280 (71.2)	0.004
	40–60	43 (20.0)	172 (80.0)	
	More than 60	7 (14.9)	40 (85.1)	
Gender	Male	74 (26.6)	204 (73.4)	0.378
	Female	89 (23.6)	288 (76.4)	
Education	High school	18 (20.2)	71 (79.8)	0.373
	Diploma and other certificates	43 (22.9)	145 (77.1)	
	Bachelors	93 (28.5)	233 (71.5)	
	PhD/Master	9 (17.3)	43 (82.7)	
Governorate	Ahmadi	26 (34.2)	50 (65.8)	0.011
	Al Asimah	25 (15.2)	140 (84.8)	
	Farwaniya	22 (31.4)	48 (68.6)	
	Hawally	36 (27.1)	97 (72.9)	
	Jahra	27 (29.0)	66 (71.0)	
	Mubarak Al Kabeer	27 (22.9)	91 (77.1)	
Following up in primary care clinics	No	69 (34.7)	130 (65.3)	<0.001
	Yes	94 (20.6)	362 (79.4)	
The primary health care center is the first choice to treat a medical problem	No	51 (61.4)	32 (38.6)	<0.001
	Yes	112 (19.6)	460 (80.4)	
Whichever is more important	Emergency department	122 (35.1)	226 (64.9)	<0.001
	Primary health care center	41 (13.4)	266 (86.6)	

The proportions of patients who selected primary care centers as their first choice for treating a medical problem in the group that considered the emergency department to be more critical and the one that regarded primary care centers as more important were not significantly different (*p* = 0.316). However, the proportions of patients who selected the emergency department as their first choice for treating a medical problem were significantly different between the groups (*p* < 0.001).

The unadjusted odds ratios (ORs) for the variables significantly associated with good impressions and good awareness are shown in Table 4. Participants who were followed-up in primary care clinics had a 1.49 times higher prevalence of good impressions than those who were not (OR = 1.49; 95% CI: 1.07–2.09, *p* = 0.018). Participants who believed that the primary health care center should be the first choice for treating a medical problem were significantly more likely to have positive views of primary health care (OR = 3.63; 95% CI: 2.19–6.00, *p* < 0.001) than those who did not. Participants who acknowledged that the primary health care center is more important than the emergency department had 2.63 times higher good impressions for primary care than those who did not (OR = 2.63; 95% CI: 1.91–3.61, *p* < 0.001).

**Table 4:** Univariable logistic regression analysis of the factors associated with good impressions and good awareness.

Good impressions				
Characteristic	*n* (%)	OR	[95% CI]	*p*
All	365 (55.7)			
Following up in primary care clinics				0.018
No	97 (48.7)	1.00	[Reference]	
Yes	268 (58.8)	1.49	[1.07–2.09]	
The primary health care center is the first option for treating a medical problem				<0.001
No	24 (28.9)	1.00	[Reference]	
Yes	341 (59.6)	3.63	[2.19–6.00]	
Whichever is more important				<0.001
Emergency department	156 (44.8)	1.00	[Reference]	
Primary health care center	209 (68.1)	2.63	[1.91–3.61]	

Good awareness				
Characteristic	*n* (%)	OR	[95% CI]	*p*
All	492 (75.1)			
Age (Years)				0.016
20–39	113 (28.8)	1.00	[Reference]	
40–60	43 (20.0)	1.60	[1.08–2.41]	
More than 60	7 (14.9)	2.30	[1.00–5.30]	
Governorate				0.013
Ahmadi	26 (34.2)	2.29	[1.24–4.25]	
Al Asimah	25 (15.2)	0.79	[0.41–1.51]	
Farwaniya	22 (31.4)	1.10	[0.61–1.99]	
Hawally	36 (27.1)	1.38	[0.74–2.56]	
Jahra	27 (29.0)	1.00	[Reference]	
Mubarak Al Kabeer	27 (22.9)	0.89	[0.46–1.75]	
Following up in primary care clinics				<0.001
No	69 (34.7)	1.00	[Reference]	
Yes	94 (20.6)	2.04	[1.41–2.96]	
The primary health care center is the first option for treating a medical problem				<0.001
No	51 (61.4)	1.00	[Reference]	
Yes	112 (19.6)	6.55	[4.02–10.66]	
Whichever is more important				<0.001
Emergency department	122 (35.1)	1.00	[Reference]	
Primary health care center	41 (13.4)	3.50	[2.36–5.20]	

OR, unadjusted odds ratio; CI, confidence interval.

After categorizing participants’ awareness scores, 492 (75.1%) had good awareness of the primary care center’s role (Table 4). Older participants (40–60 and >60 years old) had significantly higher awareness (*p* < 0.05) compared to the younger age group (20–39 years old), with odds ratios of 2.30 and 1.60, respectively. Ahmadi governorate had the highest odds of good awareness among other governorates when compared with Jahra governorate as a reference group (OR = 2.29). Participants who were followed up in primary care clinics had a 2.04 times greater prevalence of good awareness than those who were not (OR = 2.04; 95% CI: 1.41–2.96, *p* < 0.001). Participants who considered the primary health care center as the first option for medical treatment had a much higher likelihood of having positive awareness of primary health care (OR = 6.55; 95% CI: 4.02–10.66, *p* < 0.001) than those who did not. Participants who acknowledged that the primary health care center is more important than the emergency department had 2.63 times higher awareness of primary care than those who did not (OR = 3.50; 95% CI: 2.36–5.20, *p* < 0.001).

Table 5 presents the final two multivariable logistic regression models of the factors significantly and independently associated with good impressions and good awareness in the study sample. After adjusting for age, gender, education, governorate, and follow-up status in primary care clinics, participants who acknowledged that the primary health care center is the first option for treating a medical problem were significantly more likely to have good impressions and good awareness (AOR = 2.98; 95% CI: 1.77–5.02; *p* < 0.001) and (AOR = 5.09; 95% CI: 2.98–8.69; *p* < 0.001), respectively. Similarly, those who believe that the primary health care center is more important than the emergency department were more likely to have good impressions and awareness (AOR = 2.34; 95% CI: 1.68–3.25; *p* < 0.001) and (AOR = 2.64; 95% CI: 1.72–4.04; *p* < 0.001), respectively.

**Table 5:** Multivariable logistic regression model of the factors associated with good impressions and good awareness.

Characteristic	Good impressions			Good awareness		
	AOR*	[95% CI]	*p*	AOR*	[95% CI]	*p*
The primary health care center is the first option for treating a medical problem			<0.001			<0.001
No	1.00	[Reference]		1.00	[Reference]	
Yes	2.98	[1.77–5.02]		5.09	[2.98–8.69]	
Whichever is more important			<0.001			<0.001
Emergency department	1.00	[Reference]		1.00	[Reference]	
Primary health care center	2.34	[1.68–3.25]		2.64	[1.72–4.04]	

*Adjusted for age, gender, education, governorate, and follow-up status in primary care clinics. AOR, adjusted odds ratio; CI, confidence interval.

## 4 Discussion

The results of this study showed that patients who were regularly followed up at primary care centers had a higher impression of their visits to primary care doctors. Furthermore, good awareness of the role of primary care centers was correlated with higher impression scores of earlier visits to primary care doctors (*p* < 0.001). Although 78% of the participants chose primary care as their first consultation, they viewed it as less important than emergency departments. These findings were consistent with those of previous studies (12, 15, 16).

Participants who regularly visited primary care centers had higher impression scores regarding their previous visits to primary care doctors than those who did not (*p* = 0.001). Additionally, a good impression was significantly correlated with good awareness of the role of primary care centers (*p* < 0.001). These results were particularly notable among patients with chronic conditions, similar to the results of previous studies (1, 8). We found that 70.3% of the participants preferred to seek primary care for hypertension (Table 6). This preference could be attributed to the continuous doctor-patient relationship and specialized care provided by chronic disease clinics, leading to better health outcomes and higher patient satisfaction.

**Table 6:** Responses regarding the preferred specialized department for specific cases or complaints.

Case description	Primary care centers	Emergency department	Another specialized clinic
Painful urination, cases (%)	229 (38.3)	173 (28.9)	207 (34.6)
Severe headache, cases (%)	391 (64.3)	130 (21.4)	99 (16.3)
Chest pain, cases (%)	174 (28.7)	301 (49.7)	144 (23.8)
Body rash with buffy lips and shortness of breath, cases (%)	168 (27.9)	316 (52.4)	127 (21.1)
Flank pain, cases (%)	238 (39.6)	225 (42.9)	116 (19.3)
Asthma attack, cases (%)	297 (49.1)	232 (38.3)	90 (14.9)
Road traffic accident, cases (%)	53 (8.9)	469 (78.8)	87 (14.6)
Antenatal care/ contraception, cases (%)	159 (27.1)	48 (8.2)	394 (67.1)
Fever, body aches, sore throat, and cough, cases (%)	515 (83.5)	64 (10.4)	51 (8.3)
Painful one leg swelling, cases (%)	242 (40.1)	213 (35.3)	167 (27.6)
Full hand burn with blisters, cases (%)	107 (17.8)	382 (63.6)	131 (21.8)
Counseling on chronic diseases (i.e., hypertension), cases (%)	431 (70.3)	47 (7.7)	155 (25.3)
Baby with diaper rash, cases (%)	454 (76.8)	50 (8.5)	104 (17.6)
Vertigo and lightheadedness, cases (%)	498 (82.3)	62 (10.2)	62 (10.2)
A dog bite on the hand, cases (%)	154 (25.4)	399 (65.8)	70 (11.6)
Anxiety and insomnia, cases (%)	409 (67.8)	42 (7.0)	173 (28.7)
Smoke inhalation after a house fire, cases (%)	131 (21.8)	425 (70.6)	60 (10.0)
Diarrhea and vomiting for 2 days, cases (%)	389 (63.9)	170 (27.9)	69 (11.3)
A 2-week-old baby with jaundice, cases (%)	180 (30.3)	246 (41.4)	186 (31.3)
Daily wound dressing, cases (%)	557 (91.5)	38 (6.2)	25 (4.1)

Finally, 87% of the participants selected primary care as the first option for treating medical problems because emergency departments typically require a referral from a primary care doctor; thus, people tend to visit primary care centers first. However, 53% of participants perceived the emergency department as more important than primary care facilities. This perception likely reflects greater patient trust in emergency services, attributed to the continuous availability of diagnostic investigations and treatment options (5, 11, 13). In 2015, a study examining doctor-patient relationship patterns in Kuwaiti primary care centers within the context of local culture revealed a predominantly negative public image of primary care services. This unfavorable perception is rooted in widespread beliefs that primary care physicians are less competent clinicians who entered the specialty reluctantly, often motivated by lifestyle considerations rather than professional aspiration. Consequently, they are perceived as less motivated and as providers of lower-quality care—a perception shared by patients and healthcare professionals. Therefore, many primary care physicians report feeling undervalued and disrespected, frequently regarded as “second-class” when compared with hospital-based specialists, and lacking the social prestige and professional authority associated with other medical disciplines. This perception of primary care as a less legitimate component of the healthcare system has influenced utilization patterns, with many attending patients using them primarily to obtain sick leave certificates or to stockpile medications, further undermining public trust and perpetuating the idea that primary care physicians are unprofessional and undeserving of confidence.

One of the limitations of this study was the large number of incomplete responses (446), resulting in a low response rate that affected the power of the study.

In conclusion, the results of our study indicate that frequent visitors to primary care centers have a positive impression and good awareness of their role. However, a significant percentage of respondents still believed that the emergency department was more important than primary care centers. To address this issue, awareness campaigns on the role of family medicine in Kuwait are needed to improve public understanding, enhance the reputation of primary care givers, and reduce unnecessary referrals to emergency departments.

## Data Availability

The raw data supporting the conclusions of this article will be made available by the authors, without undue reservation.
